# One-Dimensional and Two-Dimensional Analytical Solutions for Functionally Graded Beams with Different Moduli in Tension and Compression

**DOI:** 10.3390/ma11050830

**Published:** 2018-05-17

**Authors:** Xue Li, Jun-yi Sun, Jiao Dong, Xiao-ting He

**Affiliations:** 1 School of Civil Engineering, Chongqing University, Chongqing 400045, China; lixuecqu@126.com (X.L.); dongjiaocqu@126.com (J.D.); hexiaoting@cqu.edu.cn (X.H.); 2 Key Laboratory of New Technology for Construction of Cities in Mountain Area (Chongqing University), Ministry of Education, Chongqing 400045, China

**Keywords:** functionally graded beams, different moduli in tension and compression, bimodulus, analytical solution, neutral layer

## Abstract

The material considered in this study not only has a functionally graded characteristic but also exhibits different tensile and compressive moduli of elasticity. One-dimensional and two-dimensional mechanical models for a functionally graded beam with a bimodular effect were established first. By taking the grade function as an exponential expression, the analytical solutions of a bimodular functionally graded beam under pure bending and lateral-force bending were obtained. The regression from a two-dimensional solution to a one-dimensional solution is verified. The physical quantities in a bimodular functionally graded beam are compared with their counterparts in a classical problem and a functionally graded beam without a bimodular effect. The validity of the plane section assumption under pure bending and lateral-force bending is analyzed. Three typical cases that the tensile modulus is greater than, equal to, or less than the compressive modulus are discussed. The result indicates that due to the introduction of the bimodular functionally graded effect of the materials, the maximum tensile and compressive bending stresses may not take place at the bottom and top of the beam. The real location at which the maximum bending stress takes place is determined via the extreme condition for the analytical solution.

## 1. Introduction

Most materials may exhibit different elastic responses in a state of tension and compression, but these characteristics are often neglected due to the complexity of their analysis. Materials that have apparently different moduli in tension and compression are known as bimodular materials [1], for example, ceramics, graphite, concrete, and some biological materials (nacre, for example [2]). During recent decades, many studies have described useful material models for studying bimodular materials. One is Bert’s model [3] based on the criterion of positive-negative signs of the strains in longitudinal fibers. This model is widely used in laminated composites [4,5,6,7,8]. Another is Ambartsumyan’s bimodular model [9] for isotropic materials, which has attracted the most attention in the engineering community. This model assesses different moduli in terms of tension and compression based on the positive-negative signs of principal stresses, which is especially important for the analysis and design of structures. It is well-known that the cracking direction of a concrete beam is always normal to the direction of principal tensile stresses in the beam. The difficulty in applying Ambartsumyan’s bimodular model is that the stress state of a point must be known in advance. However, with the exception of some fundamental problems, we must resort to finite element analysis to acquire the states of the stresses in a structure [10,11,12,13,14].

In addition to the bimodular effect in materials, it is also interesting to consider the functionally graded characteristic of materials. Functionally graded materials (FGMs) possess properties that vary gradually with the location within the material. The use of FGMs has many advantages in aerospace, automotive, and biomedical applications. There are many approximations that may be used to model the variation of properties in FGMs. One is the exponential variation, where the elastic constants vary according to the form of the exponential function. Many researchers have found this functional form to be convenient in solving elasticity problems. Sankar [15] obtained an elasticity solution for a functionally graded beam subjected to transverse loads in which the Young’s modulus is assumed to vary exponentially through the thickness and the Poisson ratio is held constant. Sankar and co-workers studied the relative issues of functionally graded beams, including thermal stresses [16], a sandwich beam with a functionally graded core [17], and a combined Fourier series–Galerkin method [18]. Without specifying the gradient variations of a material property, Zhong and co-workers presented a general solution of a functionally graded beam by the Airy stress function method [19] and a displacement function approach [20]. Daouadji et al. [21] employed the stress function approach to study the problem of a functionally graded cantilever beam subjected to a linearly distributed load, in which the Young’s modulus along the thickness direction varies with power-law functions or with exponential functions. Considering that there are many research works in this field, we do not review them in detail.

Recently, analytical studies of bimodular beams and plates have been performed. Among these works, the determination of the unknown neutral layer is a key issue because it opens up the possibility for the establishment of a mechanical model based on a subarea in tension and compression. Under the assumption that shearing stresses have no contribution to the neutral axis, Yao and Ye [22] obtained a one-dimensional analytical solution of a bimodular shallow beam. He et al. adopted the stress function method to find the elasticity solution of a bimodular straight beam [23] and curved beams [24]. Later, the classical Kirchhoff hypothesis was used to assess the existence of the elastic neutral layers of a thin plate during bending with a small deflection [25]. Consequently, a series of analytical solutions of plates is derived in rectangular and polar coordinate systems. More recently, He et al. [26] presented an elasticity solution of a bimodular FGM beam under uniformly distributed loads and discussed several concrete numerical examples. However, some basic problems are still unclear, which include the consistency between a one-dimensional solution and a two-dimensional solution, the validity of the plane section assumption, the corresponding relation among a classical beam, a standard FGM beam, and a bimodular FGM beam, as well as the bimodular effect on stresses and deformations in a general sense.

In this study, we will adopt a bimodular FGM beam theory to derive the one-dimensional and two-dimensional solutions. Theoretically speaking, any FGM beams may be suitable for this theory provided that the bimodular effect in tension and compression needs to be emphasized for a refined analysis; or, in other words, a certain constituent that forms functionally graded materials presents a relatively obvious bimodular effect which can not be ignored otherwise it will introduce much error into the analysis. The article is organized as follows. The corresponding analytical solutions under pure bending and lateral-force bending will be obtained in Section 2 and Section 3, respectively. Specifically, a perturbation method is adopted to solve the transcendental equation for the determination of the unknown neutral layer. The validity of the plane section assumption is discussed and some important physical quantities among a classical beam, a standard FGM beam, and a bimodular FGM beam are compared in Section 4. Besides this, without specifying the real magnitude of the external load and the geometrical dimension of the beam, the bimodular effect on the stress and deformation in a general sense will be investigated in Section 4. Some important conclusions and subsequent studies are given in the concluding remarks.

## 2. Functionally Graded Beams under Pure Bending

### 2.1. One-Dimensional Solution

#### 2.1.1. Bending Stress

**Figure 1:**
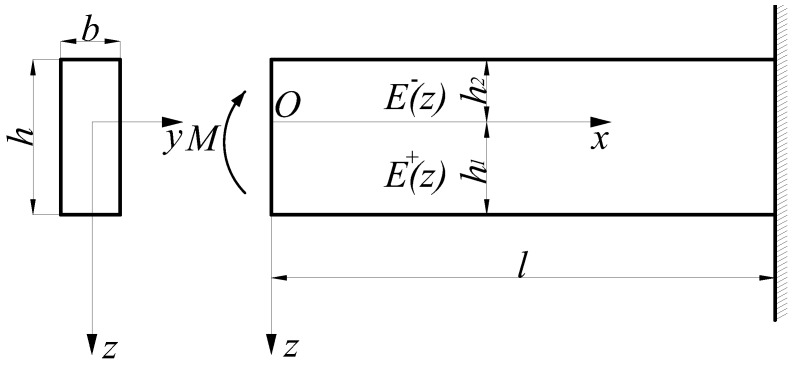
Scheme of a bimodular functionally graded material (FGM) beam under pure bending.

A bimodular functionally graded beam with a rectangular section dimension of *h* × *b* is subjected to a bending moment *M* alone as shown in Figure 1. This causes a bending of the beam in the plane coordinate system *xoz*. Note that due to the introduction of the bimodular effect in tension and compression as well as the functionally graded characteristic of the material, the neutral layer of the beam generally does not locate on the half height of the section. The *x* axis is established on the unknown neutral layer as shown in Figure 1. It is obvious that the zone below the neutral layer is in tension while the zone up the layer is in compression. Let the tensile and compressive section heights of the beam be $h_{1}$ and $h_{2}$, respectively. Also, let the modulus of elasticity of the material in the tensile and compressive zones be $E^{+}(z)$ and $E^{-}(z)$, respectively, while the Poisson’s ratios remain the same.

If an exponential function is used to express the function grade of the material, $E^{+}(z)$ and $E^{-}(z)$ may be expressed as

(1) $$E^{+}(z)=E_{0}e^{\alpha_{1}z/h},E^{-}(z)=E_{0}e^{\alpha_{2}z/h},$$

where $\alpha_{1}$ and $\alpha_{2}$ are two grade indexes. $E^{+}(z)=E^{-}(z)=E_{0}$ when z=0, that is, at the neutral layer the tensile modulus is equal to the compressive one. Let the curvature radius of the neutral layer be ρ; then, the bending strain along the *x* axis in the whole beam will be the uniform expression $\varepsilon_{x}=z/\rho$ if the plane section assumption holds. Thus, according to Ambartsumyan’s bimodular model the bending stress in the tensile and compressive zones, $\sigma_{x}^{+}$ and $\sigma_{x}^{-}$, are also the tensile and compressive principal stress and they are, respectively,

(2) $$\sigma_{x}^{+}=\frac{E^{+}(z)}{\rho }z,\;\mathrm{for}\;0\leq z\leq h_{1},$$

and

(3) $$\sigma_{x}^{-}=\frac{E^{-}(z)}{\rho }z,\;\mathrm{for}\;-h_{2}\leq z\leq 0.$$

Let the normal resultant at any section be *N*, thus N=0 yields

(4) $$\int_{0}^{h_{1}}\sigma_{x}^{+}bdz+\int_{-h_{2}}^{0}\sigma_{x}^{-}bdz=0.$$

Substituting Equations (2) and (3) into Equation (4), we have

(5) $$\int_{0}^{h_{1}}\frac{E_{0}e^{\alpha_{1}z/h}zb}{\rho }dz+\int_{-h_{2}}^{0}\frac{E_{0}e^{\alpha_{2}z/h}zb}{\rho }dz=0.$$

If we let

(6) $$\begin{array}{l} \int_{0}^{h_{1}}e^{\alpha_{1}z/h}zdz=\left(\frac{hh_{1}}{\alpha_{1}}-\frac{h^{2}}{\alpha_{1}^{2}}\right)e^{\alpha_{1}h_{1}/h}+\frac{h^{2}}{\alpha_{1}^{2}}=A_{1}^{+}\\ \int_{-h_{2}}^{0}e^{\alpha_{2}z/h}zdz=\left(\frac{hh_{2}}{\alpha_{2}}+\frac{h^{2}}{\alpha_{2}^{2}}\right)e^{-\alpha_{2}h_{2}/h}-\frac{h^{2}}{\alpha_{2}^{2}}=A_{1}^{-} \end{array},$$

Equation (4) will lead to the following relation

(7) $$A_{1}^{+}+A_{1}^{-}=0,$$

which is used for the determination of the unknown neutral layer later.

Similarly, the bending moment at any section will give

(8) $$\int_{0}^{h_{1}}\sigma_{x}^{+}bzdz+\int_{-h_{2}}^{0}\sigma_{x}^{-}bzdz=M.$$

Substituting Equations (2) and (3) into Equation (8), we have

(9) $$\int_{0}^{h_{1}}\frac{E_{0}e^{\alpha_{1}z/h}z^{2}b}{\rho }dz+\int_{-h_{2}}^{0}\frac{E_{0}e^{\alpha_{2}z/h}z^{2}b}{\rho }dz=M.$$

If we let

(10) $$\begin{array}{l} \int_{0}^{h_{1}}e^{\alpha_{1}z/h}z^{2}dz=\left(\frac{hh_{1}^{2}}{\alpha_{1}}-2\frac{h^{2}h_{1}}{\alpha_{1}^{2}}+2\frac{h^{3}}{\alpha_{1}^{3}}\right)e^{\alpha_{1}h_{1}/h}-2\frac{h^{3}}{\alpha_{1}^{3}}=A_{2}^{+}\\ \int_{-h_{2}}^{0}e^{\alpha_{2}z/h}z^{2}dz=-\left(\frac{hh_{2}^{2}}{\alpha_{2}}+2\frac{h^{2}h_{2}}{\alpha_{2}^{2}}+2\frac{h^{3}}{\alpha_{2}^{3}}\right)e^{-\alpha_{2}h_{2}/h}+2\frac{h^{3}}{\alpha_{2}^{3}}=A_{2}^{-} \end{array},$$

Equation (8) will yield

(11) $$\frac{1}{\rho }=\frac{M}{E_{0}b(A_{2}^{+}+A_{2}^{-})}.$$

If $D^{*}$ is introduced to denote the flexural stiffness of the bimodular functionally graded beam, that is,

(12) $$D^{*}=E_{0}b(A_{2}^{+}+A_{2}^{-}),$$

the deformation of the beam will follow the familiar form

(13) $$\frac{1}{\rho }=\frac{M}{D^{*}}.$$

Substituting the relation (11) into Equations (2) and (3), we obtain the one-dimensional solution of the bending stress in the tensile and compressive zones, respectively,

(14) $$\sigma_{x}^{+}=\frac{Me^{\alpha_{1}z/h}z}{b(A_{2}^{+}+A_{2}^{-})},\;\mathrm{for}\;0\leq z\leq h_{1},$$

and

(15) $$\sigma_{x}^{-}=\frac{Me^{\alpha_{2}z/h}z}{b(A_{2}^{+}+A_{2}^{-})},\;\mathrm{for}\;-h_{2}\leq z\leq 0.$$

It should be noted here that due to this being the pure bending case, only the bending stress may be obtained and the shearing stress can be derived in the lateral-force bending case, which will be discussed in Section 3.

#### 2.1.2. Deflection Curve

Let the vertical displacement of any point on the neutral layer be *w*; then, Equation (11) may be expressed in terms of the second-order derivative of *w* to *x* as follows

(16) $$\frac{1}{\rho }=-\frac{d^{2}w}{dx^{2}}=\frac{M}{E_{0}b(A_{2}^{+}+A_{2}^{-})}.$$

Integrating twice with respect to *x*, we have

(17) $$w=-\frac{Mx^{2}}{2E_{0}b(A_{2}^{+}+A_{2}^{-})}+cx+d,$$

where *c* and *d* are two undetermined constants. If a simply-supported beam is considered, the boundary conditions give

(18) w=0, while x=0 or l,

where *l* is the span length of the beam. Thus, the deflection curve of the neutral axis is

(19) $$w=\frac{M(l-x)x}{2E_{0}b(A_{2}^{+}+A_{2}^{-})}.$$

If a cantilever beam with the right end fixed is considered, as shown in Figure 1, the displacement restriction is

(20) $$w=\frac{dw}{dx}=0,\;\mathrm{while}\;x=l,$$

and the deflection curve of the neutral axis will be

(21) $$w=-\frac{M{\left(x-l\right)}^{2}}{2E_{0}b(A_{2}^{+}+A_{2}^{-})}.$$

#### 2.1.3. Determination of the Neutral Layer

It should be noted here that the two important parameters $h_{1}$ and $h_{2}$ have still not been determined. From Equations (6) and (7), we may have

(22) $$\left(\frac{h_{1}}{\alpha_{1}}-\frac{h}{\alpha_{1}^{2}}\right)e^{\alpha_{1}h_{1}/h}+\left(\frac{h_{2}}{\alpha_{2}}+\frac{h}{\alpha_{2}^{2}}\right)e^{-\alpha_{2}h_{2}/h}=\frac{h}{\alpha_{2}^{2}}-\frac{h}{\alpha_{1}^{2}},$$

where $\alpha_{1}$ and $\alpha_{2}$ are two indexes concerning the grade function as indicated above. If we introduce the following dimensionless variables

(23) $$H_{1}=\frac{h_{1}}{h},\;H_{2}=\frac{h_{2}}{h},$$

and also multiply the two ends of the equation by $\alpha_{1}^{2}\alpha_{2}^{2}$, Equation (22) may be transformed into a dimensionless form, such that

(24) $$(\alpha_{1}H_{1}-1)\alpha_{2}^{2}e^{\alpha_{1}H_{1}}+(\alpha_{2}H_{2}+1)\alpha_{1}^{2}e^{-\alpha_{2}H_{2}}=\alpha_{1}^{2}-\alpha_{2}^{2},$$

in which $H_{1}$ and $H_{2}$ are the basic variables and satisfy $H_{1}+H_{2}=1$. It is a transcendental equation and is hard to solve analytically to some extent due to the existence of an exponential function. Next, we will adopt the perturbation idea to solve the transcendental equation.

The exponential items $e^{\alpha_{1}H_{1}}$ and $e^{-\alpha_{2}H_{2}}$ may be spread with respect to $H_{1}$ and $H_{2}$, respectively,

(25) $$\begin{array}{l} e^{\alpha_{1}H_{1}}=1+\alpha_{1}H_{1}+\frac{1}{2}\alpha_{1}^{2}H_{1}^{2}+\cdots +\frac{1}{n!}{\left(\alpha_{1}H_{1}\right)}^{n}+\cdots ,\\ e^{-\alpha_{2}H_{2}}=1-\alpha_{2}H_{2}+\frac{1}{2}\alpha_{2}^{2}H_{2}^{2}+\cdots +\frac{1}{n!}{\left(-\alpha_{2}H_{2}\right)}^{n}+\cdots . \end{array}$$

If the linear approximation is adopted, such that

(26) $$e^{\alpha_{1}H_{1}}=1+\alpha_{1}H_{1},\;e^{-\alpha_{2}H_{2}}=1-\alpha_{2}H_{2},$$

substituting it into Equation (24) will yield

(27) $$H_{1}=H_{2}=\frac{1}{2},$$

which is exactly the solution of a classical problem without considering the functionally graded property and bimodular effect of the material. We call it the first-order approximation solution of the problem. If the second-order approximation is adopted, such that

(28) $$e^{\alpha_{1}H_{1}}=1+\alpha_{1}H_{1}+\frac{1}{2}\alpha_{1}^{2}H_{1}^{2},\;e^{-\alpha_{2}H_{2}}=1-\alpha_{2}H_{2}+\frac{1}{2}\alpha_{2}^{2}H_{2}^{2},$$

substituting it into Equation (24) and considering $H_{2}=1-H_{1}$ yields

(29) $$(\alpha_{1}-\alpha_{2})H_{1}^{3}+3\alpha_{2}H_{1}^{2}+(2-3\alpha_{2})H_{1}+\alpha_{2}-1=0,$$

which is an algebra equation of $H_{1}$ and is easily solved either by an analytical method or by a numerical technique once the numerical values of $\alpha_{1}$ and $\alpha_{2}$ are known. The solution of Equation (29) may be called the second-order approximation solution. Similarly, if more items in Equation (25) are taken, we will obtain a high-order approximation solution according to the procedure indicated above. Thus, based on the perturbation idea, the transcendental equation may be gradually transformed into a nonlinear algebra equation of $H_{1}$ and the position of the unknown neutral layer is determined analytically.

### 2.2. Two-Dimensional Solution

#### 2.2.1. Stress

Let the stress components in the two-dimensional beam problem shown in Figure 1 be $\sigma_{x}$, $\sigma_{z}$, and $\tau_{xz}$, let the strain components be $\varepsilon_{x}$, $\varepsilon_{z}$, and $\gamma_{xz}$, and also let the displacement components in the same problem be *u* and *w*. Then, in the differential equation of equilibrium in which the body forces are neglected, the geometrical relation as well as the consistency equation are the same as those in the classical problem, and they are, respectively,

(30) $$\frac{\partial \sigma_{x}}{\partial x}+\frac{\partial \tau_{xz}}{\partial z}=0,\frac{\partial \tau_{zx}}{\partial x}+\frac{\partial \sigma_{x}}{\partial z}=0,$$

and

(31) $$\left\{ \begin{array}{l} \varepsilon_{x}=\frac{\partial u}{\partial x},\;\varepsilon_{z}=\frac{\partial w}{\partial z},\;\gamma_{xz}=\frac{\partial w}{\partial x}+\frac{\partial u}{\partial z}\\ \frac{\partial^{2}\varepsilon_{x}}{\partial z^{2}}+\frac{\partial^{2}\varepsilon_{z}}{\partial x^{2}}=\frac{\partial^{2}\gamma_{xz}}{\partial x\partial z} \end{array}\right..$$

The physical equation gives

(32) $$\left\{ \begin{array}{l} \varepsilon_{x}=s_{11}\sigma_{x}+s_{13}\sigma_{z}\\ \varepsilon_{z}=s_{13}\sigma_{x}+s_{33}\sigma_{z}\\ \gamma_{zx}=s_{44}\tau_{zx} \end{array}\right..$$

After considering the different moduli in tension and compression as well as the functional grade of the material, the physical equation may take the following form

(33) $$\left\{ \begin{array}{l} \varepsilon_{x}^{+/-}=\frac{1}{E_{0}e^{\alpha_{i}z/h}}(\sigma_{x}^{+/-}-\mu \sigma_{z}^{+/-})\\ \varepsilon_{z}^{+/-}=\frac{1}{E_{0}e^{\alpha_{i}z/h}}(\sigma_{z}^{+/-}-\mu \sigma_{x}^{+/-})\\ \gamma_{zx}^{+/-}=\frac{2(1+\mu )}{E_{0}e^{\alpha_{i}z/h}}\tau_{zx}^{+/-} \end{array}\right.,$$

where a superscript “+/−” denotes a tensile (compressive) quantity and $\alpha_{i}(i=1,2)$ correspond to the cases of tension and compression, respectively. Equation (33) is in essence two sets of equations concerning tension and compression.

Next, the stress function method will be adopted to obtain the solution of this two-dimensional problem. Due to pure bending, here we still consider that the stress function $\varphi^{+/-}(x,z)$ depends only on *z*, that is

(34) $$\varphi^{+/-}(x,z)=f^{+/-}(z),$$

where $f^{+/-}(z)$ is an unknown function and “+/−” still denotes a tensile (compressive) quantity. According to the relation between the stress function and the stress components,

(35) $$\sigma_{x}^{+/-}=\frac{\partial^{2}\varphi^{+/-}}{\partial z^{2}},\;\sigma_{z}^{+/-}=\frac{\partial^{2}\varphi^{+/-}}{\partial x^{2}},\;\tau_{xz}^{+/-}=-\frac{\partial^{2}\varphi^{+/-}}{\partial x\partial z}.$$

Equation (33) may be changed as

(36) $$\left\{ \begin{array}{l} \varepsilon_{x}^{+/-}=\frac{1}{E_{0}e^{\alpha_{i}z/h}}\frac{d^{2}f^{+/-}(z)}{dz^{2}}\\ \varepsilon_{z}^{+/-}=\frac{-\mu }{E_{0}e^{\alpha_{i}z/h}}\frac{d^{2}f^{+/-}(z)}{dz^{2}}\\ \gamma_{zx}^{+/-}=0 \end{array}\right..$$

Letting Equation (36) satisfy the consistency relation, we obtain

(37) $$\frac{d^{2}}{dz^{2}}\left[\frac{1}{E_{0}e^{\alpha_{i}z/h}}\frac{d^{2}f^{+/-}(z)}{dz^{2}}\right]=0.$$

Integrating twice with respect to *z*, we have

(38) $$\frac{d^{2}f^{+/-}(z)}{dz^{2}}=(C_{1}^{+/-}z+C_{2}^{+/-})E_{0}e^{\alpha_{i}z/h},$$

where $C_{1}^{+/-}$ and $C_{2}^{+/-}$ are four undetermined constants. Continuously integrating with respect to *z*, we obtain

(39) $$f^{+/-}(z)=\left(z-\frac{2h}{\alpha_{i}}\right)\frac{E_{0}C_{1}^{+/-}h^{2}e^{\alpha_{i}z/h}}{\alpha_{i}^{2}}+\frac{E_{0}C_{2}^{+/-}h^{2}e^{\alpha_{i}z/h}}{\alpha_{i}^{2}}+C_{3}^{+/-}z+C_{4}^{+/-},$$

where $C_{3}^{+/-}$ and $C_{4}^{+/-}$ are four undetermined constants and may be neglected. The stress function is simplified as

(40) $$\varphi^{+/-}(x,z)=\left(z-\frac{2h}{\alpha_{i}}\right)\frac{E_{0}C_{1}^{+/-}h^{2}e^{\alpha_{i}z/h}}{\alpha_{i}^{2}}+\frac{E_{0}C_{2}^{+/-}h^{2}e^{\alpha_{i}z/h}}{\alpha_{i}^{2}}.$$

Correspondingly, the stress expressions are

(41) $$\sigma_{x}^{+/-}=(C_{1}^{+/-}z+C_{2}^{+/-})E_{0}e^{\alpha_{i}z/h},\sigma_{z}^{+/-}=0,\;\tau_{zx}^{+/-}=0.$$

Next, we will use the boundary conditions as well as the continuity condition of stress to determine the four unknown constants $C_{1}^{+/-}$ and $C_{2}^{+/-}$.

First, the continuity conditions of the stresses on the neutral layer give

(42) $$\sigma_{x}^{+}=\sigma_{x}^{-}=0,\;\sigma_{z}^{+}=\sigma_{z}^{-},\;\tau_{xz}^{+}=\tau_{xz}^{-}\;\mathrm{at}\;z=0.$$

According to Equation (41), it is easily found that the last two conditions are surely satisfied and the first condition yields

(43) $$C_{2}^{+}=C_{2}^{-}=0.$$

The stress boundary conditions on the two main sides of the beam are, respectively,

(44) $$\left\{ \begin{array}{l} \sigma_{z}^{+}=0,\;\tau_{xz}^{+}=0\;\mathrm{at}\;z=h_{1}\\ \sigma_{z}^{-}=0,\;\tau_{xz}^{-}=0\;\mathrm{at}\;z=-h_{2} \end{array}\right.,$$

which are surely satisfied due to pure bending. At the left end of the beam, the application of Saint-Venant’s Principle gives

(45) $$\left\{ \begin{array}{l} \int_{0}^{h_{1}}\sigma_{x}^{+}bdz+\int_{-h_{2}}^{0}\sigma_{x}^{-}bdz=0,\\ \int_{0}^{h_{1}}\sigma_{x}^{+}zbdz+\int_{-h_{2}}^{0}\sigma_{x}^{-}zbdz=M\\ \int_{0}^{h_{1}}\tau_{xz}^{+}bdz+\int_{-h_{2}}^{0}\tau_{xz}^{-}bdz=0, \end{array}\right.,\;\mathrm{at}\;x=0.$$

It is easily found that the last condition is satisfied and the first two conditions will yield, respectively,

(46) $$C_{1}^{+}\int_{0}^{h_{1}}ze^{\alpha_{1}z/h}dz+C_{1}^{-}\int_{-h_{2}}^{0}ze^{\alpha_{2}z/h}dz=0,$$

and

(47) $$C_{1}^{+}\int_{0}^{h_{1}}z^{2}e^{\alpha_{1}z/h}dz+C_{1}^{-}\int_{-h_{2}}^{0}z^{2}e^{\alpha_{2}z/h}dz=\frac{M}{E_{0}b}.$$

Considering the Equations (6), (7), and (10), we solve

(48) $$C_{1}^{+}=C_{1}^{-}=\frac{M}{E_{0}b(A_{2}^{+}+A_{2}^{-})}.$$

Thus, the final stress components are

(49) $$\sigma_{x}^{+/-}=\frac{M}{b(A_{2}^{+}+A_{2}^{-})}ze^{\alpha_{i}z/h},\sigma_{z}^{+/-}=0,\;\tau_{zx}^{+/-}=0,$$

which is the same as the one-dimensional solution obtained in Section 2.1.1.

#### 2.2.2. Displacement

After the determination of the stress components, the combination of the physical equations and the geometrical equations will give

(50) $$\left\{ \begin{array}{l} \varepsilon_{x}^{+/-}=\frac{M}{E_{0}b(A_{2}^{+}+A_{2}^{-})}z=\frac{\partial u}{\partial x}\\ \varepsilon_{z}^{+/-}=\frac{-\mu M}{E_{0}b(A_{2}^{+}+A_{2}^{-})}z=\frac{\partial w}{\partial z}\\ \gamma_{zx}^{+/-}=0=\frac{\partial u}{\partial z}+\frac{\partial w}{\partial x} \end{array}\right..$$

Integrating the first two expressions with respect to *x* and *z*, we have, respectively,

(51) $$u=\frac{M}{E_{0}b(A_{2}^{+}+A_{2}^{-})}zx+g_{1}(z),$$

and

(52) $$w=\frac{-\mu M}{2E_{0}b(A_{2}^{+}+A_{2}^{-})}z^{2}+g_{2}(x),$$

where $g_{1}(z)$ and $g_{2}(x)$ are two undermined functions. Substituting u and w into the third expression in Equation (50), we have

(53) $$\frac{M}{E_{0}b(A_{2}^{+}+A_{2}^{-})}x+\frac{dg_{2}(x)}{dx}=-\frac{dg_{1}(z)}{dz}=a,$$

where a is a rigid displacement item. Integrating the above expression with respect to *z* and *x*, we have, respectively,

(54) $$g_{1}(z)=-az+c,$$

and

(55) $$g_{2}(x)=-\frac{M}{2E_{0}b(A_{2}^{+}+A_{2}^{-})}x^{2}+ax+d,$$

where and d are still rigid displacement items. Now, the displacement may be expressed as

(56) $$u=\frac{M}{E_{0}b(A_{2}^{+}+A_{2}^{-})}zx-az+c,$$

and

(57) $$w=-\frac{M}{2E_{0}b(A_{2}^{+}+A_{2}^{-})}(x^{2}+\mu z^{2})+ax+d.$$

If we consider here a simply-supported beam, the corresponding boundary conditions give

(58) $$\left\{ \begin{array}{l} u=w=0,\;\mathrm{while}\;x=0,\;z=0\\ w=0,\;\mathrm{while}\;x=l,\;z=0 \end{array}\right.,$$

where *l* is the span length of the beam. Thus, the last displacement components are

(59) $$\left\{ \begin{array}{l} u(x,z)=\frac{M}{2E_{0}b(A_{2}^{+}+A_{2}^{-})}(2x-l)z\\ w(x,z)=-\frac{M}{2E_{0}b(A_{2}^{+}+A_{2}^{-})}(x^{2}+\mu z^{2}-lx) \end{array}\right..$$

The deflection curve of the neutral layer may be obtained by ${w(x,z)|}_{z=0}$, which is the same as the one-dimensional solution, i.e., Equation (19). If a cantilever beam with the right end fixed is considered, the restriction conditions yield

(60) $$u=w=\frac{\partial w}{\partial x}=0,\;\mathrm{while}\;x=l,\;z=0,$$

the last displacement components will be

(61) $$\left\{ \begin{array}{l} u(x,z)=\frac{M}{E_{0}b(A_{2}^{+}+A_{2}^{-})}(x-l)z\\ w(x,z)=-\frac{M}{2E_{0}b(A_{2}^{+}+A_{2}^{-})}(x^{2}+\mu z^{2}-2lx+l^{2}) \end{array}\right..$$

Similarly, the deflection curve of the neutral layer is consistent with the result presented in Equation (21).

## 3. Bimodular Functionally Graded Beams under Latera-Force Bending

**Figure 2:**
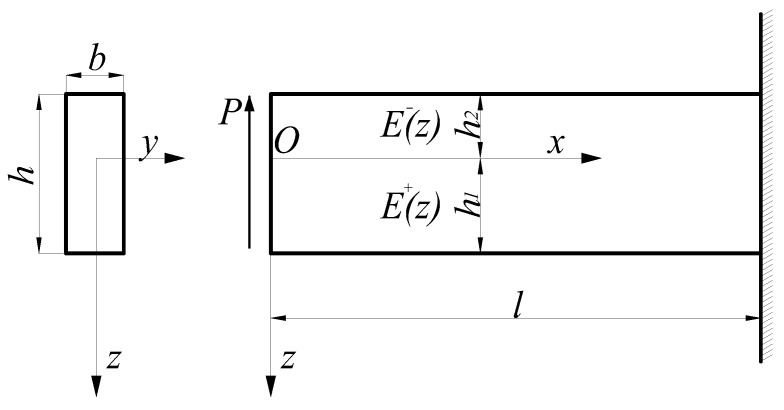
Scheme of a bimodular FGM beam under lateral-force bending.

Let us consider the lateral-force bending problem of a bimodular functionally graded beam, as shown in Figure 2, in which the left end of the beam is subjected to the action of a concentrated force *P* and the right end is fixed. Due to the combined action of the bending moment and the shearing force, any point in the beam is in diagonal tension or diagonal compression; so, it is very difficult to determine the position and shape of the unknown neutral layer if the constitutive law defined in the principal stress direction is strictly followed. For this purpose, an important assumption that shearing stresses have no contribution to the neutral axis [22] is used to establish the simplified mechanical model. In the light of the assumption, the beam will deflect and develop a so-called tensile zone and compressive zone under the external load. The tension and compression of any point in the beam depend only on the direction of the bending stress and are independent of the shearing stress. Thus, similar to the case of pure bending shown in Figure 1, the mechanical model based on a subarea in tension and compression is still established in the case of lateral-force bending as shown in Figure 2. The basic equations of the problem are the same as those in Section 2.2.1, that is, Equations (30)–(33). According to the loading conditions, the stress function may be assumed to be

(62) $$\varphi^{+/-}=xf^{+/-}(z),$$

where $f^{+/-}(z)$ is an unknown function, and it may be determined by satisfying the consistency relation. The strain components expressed in term of $f^{+/-}(z)$ are

(63) $$\left\{ \begin{array}{l} \varepsilon_{x}^{+/-}=\frac{x}{E_{0}e^{\alpha_{i}z/h}}\frac{d^{2}f^{+/-}(z)}{dz^{2}}\\ \varepsilon_{z}^{+/-}=\frac{-\mu x}{E_{0}e^{\alpha_{i}z/h}}\frac{d^{2}f^{+/-}(z)}{dz^{2}}\\ \gamma_{zx}^{+/-}=\frac{-2(1+\mu )}{E_{0}e^{\alpha_{i}z/h}}\frac{df^{+/-}(z)}{dz} \end{array}\right..$$

Satisfying the consistency relation for any *x* gives

(64) $$\frac{d^{2}}{dz^{2}}\left[\frac{1}{E_{0}e^{\alpha_{i}z/h}}\frac{d^{2}f^{+/-}(z)}{dz^{2}}\right]=0.$$

Continuously integrating with respect to *z*, we have

(65) $$f^{+/-}(z)=\left(z-\frac{2h}{\alpha_{i}}\right)\frac{E_{0}C_{1}^{+/-}h^{2}e^{\alpha_{i}z/h}}{\alpha_{i}^{2}}+\frac{E_{0}C_{2}^{+/-}h^{2}e^{\alpha_{i}z/h}}{\alpha_{i}^{2}}+C_{3}^{+/-}z,$$

where $C_{1}^{+/-},C_{2}^{+/-}\;\mathrm{and}\;C_{3}^{+/-}$ are six undetermined constants; the constant item has been neglected. Thus, the stress function now has the following form

(66) $$\varphi^{+/-}=x\left[\left(z-\frac{2h}{\alpha_{i}}\right)\frac{E_{0}C_{1}^{+/-}h^{2}e^{\alpha_{i}z/h}}{\alpha_{i}^{2}}+\frac{E_{0}C_{2}^{+/-}h^{2}e^{\alpha_{i}z/h}}{\alpha_{i}^{2}}+C_{3}^{+/-}z\right].$$

The stress components expressed in terms of the undetermined constants are

(67) $$\left\{ \begin{array}{l} \sigma_{x}^{+/-}=x(C_{1}^{+/-}z+C_{2}^{+/-})E_{0}e^{\alpha_{i}z/h},\sigma_{z}^{+/-}=0\\ \tau_{zx}^{+/-}=-(\alpha_{i}z-h)\frac{E_{0}C_{1}^{+/-}he^{\alpha_{i}z/h}}{\alpha_{i}^{2}}-\frac{E_{0}C_{2}^{+/-}he^{\alpha_{i}z/h}}{\alpha_{i}}-C_{3}^{+/-} \end{array}\right..$$

The continuity conditions of the stresses on the neutral layer under lateral-force bending are the same as those under pure bending; thus, applying Equation (42) yields

(68) $$C_{2}^{+}=C_{2}^{-}=0,$$

and

(69) $$\frac{E_{0}C_{1}^{+}h^{2}}{\alpha_{1}^{2}}-C_{3}^{+}=\frac{E_{0}C_{1}^{-}h^{2}}{\alpha_{2}^{2}}-C_{3}^{-}.$$

Similarly, the stress boundary conditions on the two main sides of the beam are the same as those in Equation (44). Satisfying the conditions in the tensile and compressive zones yields, respectively,

(70) $$\tau_{zx}^{+}=-\left(\alpha_{1}h_{1}-h\right)\frac{E_{0}C_{1}^{+}he^{\alpha_{1}h_{1}/h}}{\alpha_{1}^{2}}-C_{3}^{+}=0,$$

and

(71) $$\tau_{zx}^{-}=-\left(-\alpha_{2}h_{2}-h\right)\frac{E_{0}C_{1}^{-}he^{-\alpha_{2}h_{2}/h}}{\alpha_{2}^{2}}-C_{3}^{-}=0.$$

At the left end of the beam, the application of Saint-Venant’s Principle gives

(72) $$\left\{ \begin{array}{l} \int_{0}^{h_{1}}\sigma_{x}^{+}bdz+\int_{-h_{2}}^{0}\sigma_{x}^{-}bdz=0,\\ \int_{0}^{h_{1}}\sigma_{x}^{+}zbdz+\int_{-h_{2}}^{0}\sigma_{x}^{-}zbdz=0\\ \int_{0}^{h_{1}}\tau_{xz}^{+}bdz+\int_{-h_{2}}^{0}\tau_{xz}^{-}bdz=P, \end{array}\right.,\;\mathrm{at}\;x=0.$$

It is easily found that the first two conditions are satisfied and the last condition gives

(73) $$\int_{0}^{h_{1}}\left[\left(\alpha_{1}z-h\right)\frac{E_{0}C_{1}^{+}he^{\alpha_{1}z/h}}{\alpha_{1}^{2}}+C_{3}^{+}\right]dz+\int_{-h_{2}}^{0}\left[\left(\alpha_{2}z-h\right)\frac{E_{0}C_{1}^{-}he^{\alpha_{2}z/h}}{\alpha_{2}^{2}}+C_{3}^{-}\right]dz=-\frac{P}{b}.$$

Equations (69), (70), (71), and (73) may be used for the solution of $C_{1}^{+/-}\mathrm{and}\;C_{3}^{+/-}$. First, substituting Equations (70) and (71) into Equation (69) and also considering $A_{1}^{+}$ and $A_{1}^{+}$ introduced beforehand, we have a simple expression

(74) $$A_{1}^{+}C_{1}^{+}+A_{1}^{-}C_{1}^{-}=0,$$

which gives $C_{1}^{+}=C_{1}^{-}$ due to $A_{1}^{+}+A_{1}^{-}=0$. Second, integrating Equation (73), substituting Equations (70) and (71) into it, and also considering $A_{2}^{+}$ and $A_{2}^{-}$ introduced beforehand, Equation (73) may be simplified as

(75) $$A_{2}^{+}C_{1}^{+}+A_{2}^{-}C_{1}^{-}=\frac{P}{E_{0}b}.$$

Combining Equations (74) and (75) will solve $C_{1}^{+}$ and $C_{1}^{-}$, and substituting them into Equations (70) and (71), we finally obtain

(76) $$\left\{ \begin{array}{l} C_{1}^{+}=C_{1}^{-}=\frac{P}{E_{0}b(A_{2}^{+}+A_{2}^{-})}\\ C_{3}^{+}=\frac{Pe^{\alpha_{1}h_{1}/h}}{b(A_{2}^{+}+A_{2}^{-})}\left(\frac{h^{2}}{\alpha_{1}^{2}}-\frac{hh_{1}}{\alpha_{1}}\right),\\ C_{3}^{-}=\frac{Pe^{-\alpha_{2}h_{2}/h}}{b(A_{2}^{+}+A_{2}^{-})}\left(\frac{h^{2}}{\alpha_{2}^{2}}+\frac{hh_{2}}{\alpha_{2}}\right) \end{array}\right.$$

Substituting the determined $C_{1}^{+/-},C_{2}^{+/-}\;\mathrm{and}\;C_{3}^{+/-}$ into Equation (67), the stress components are obtained as follows

(77) $$\left\{ \begin{array}{l} \sigma_{x}^{+/-}=\frac{Px}{b(A_{2}^{+}+A_{2}^{-})}ze^{\alpha_{i}z/h},\sigma_{z}^{+/-}=0\\ \tau_{zx}^{+}=\frac{P}{b(A_{2}^{+}+A_{2}^{-})}\left[\left(\frac{h^{2}}{\alpha_{1}^{2}}-\frac{hz}{\alpha_{1}}\right)e^{\alpha_{1}z/h}-\left(\frac{h^{2}}{\alpha_{1}^{2}}-\frac{hh_{1}}{\alpha_{1}}\right)e^{\alpha_{1}h_{1}/h}\right]\\ \tau_{zx}^{-}=\frac{P}{b(A_{2}^{+}+A_{2}^{-})}\left[\left(\frac{h^{2}}{\alpha_{2}^{2}}-\frac{hz}{\alpha_{2}}\right)e^{\alpha_{2}z/h}-\left(\frac{h^{2}}{\alpha_{2}^{2}}+\frac{hh_{2}}{\alpha_{2}}\right)e^{-\alpha_{2}h_{2}/h}\right] \end{array}\right..$$

It is easily found that the item Px in $\sigma_{x}^{+/-}$ is exactly the magnitude of the bending moment, which is consistent with Equations (14) and (15).

By use of the physical equation and the geometrical equation, the displacement components may be determined as

(78) $$\left\{ \begin{array}{l} u^{+}=\frac{P[(1+\mu )h(12h\alpha_{1}z-6\alpha_{1}^{2}z^{2})+\mu \alpha_{1}^{3}z^{3}+3\alpha_{1}^{3}x^{2}z+12(1+\mu )h^{2}e^{\alpha_{1}(h_{1}-z)/h}\left(h-h_{1}\alpha_{1}\right)]}{6E_{0}b\alpha_{1}^{3}(A_{2}^{+}+A_{2}^{-})}-a^{+}z-c^{+}\\ u^{-}=\frac{P[(1+\mu )h(12h\alpha_{2}z-6\alpha_{2}^{2}z^{2})+\mu \alpha_{2}^{3}z^{3}+3\alpha_{2}^{3}x^{2}z+12(1+\mu )h^{2}e^{-\alpha_{2}(h_{2}+z)/h}(h+h_{2}\alpha_{2})]}{6E_{0}b\alpha_{2}^{3}(A_{2}^{+}+A_{2}^{-})}-a^{-}z-c^{-}\\ w^{+/-}=\frac{P}{6E_{0}b(A_{2}^{+}+A_{2}^{-})}(-3\mu xz^{2}-x^{3})+a^{+/-}x+d^{+/-} \end{array}\right.,$$

where *a, d*, and *c* are the items concerning rigid displacement. Using the boundary condition u=w=∂w/∂x=0 at x=l, z=0, we have

(79) $$\left\{ \begin{array}{l} a^{+/-}=\frac{Pl^{2}}{2E_{0}b(A_{2}^{+}+A_{2}^{-})},d^{+/-}=-\frac{Pl^{3}}{3E_{0}b(A_{2}^{+}+A_{2}^{-})},\\ c^{+}=\frac{2(1+\mu )h^{2}(h-h_{1}\alpha_{1})Pe^{\alpha_{1}h_{1}/h}}{E_{0}b\alpha_{1}^{3}(A_{2}^{+}+A_{2}^{-})},c^{-}=\frac{2(1+\mu )h^{2}(h+h_{2}\alpha_{2})Pe^{-\alpha_{2}h_{2}/h}}{E_{0}b\alpha_{2}^{3}(A_{2}^{+}+A_{2}^{-})} \end{array}\right..$$

Thus, the final displacements are determined.

## 4. Results and Discussions

### 4.1. Comparision among Three Types of Beam

As indicated before, the material considered in this study not only has a functionally graded characteristic but also presents different mechanical properties in tension and compression. It is valuable to compare physical quantities in a bimodular FGM beam and a standard FGM beam (without bimodular effect) with their counterparts in a classical problem. We should note that in a classical problem, there is no variation of material properties along the thickness direction; thus, the relevant integrals are usually done over the whole section height. The comparisons among the three types of beams are listed in Table 1. It is easily found that when the grade indexes $\alpha_{1}=\alpha_{2}$, the quantities in a bimodular FGM beam regress to the corresponding quantities in a standard FGM beam; when $\alpha_{1}=\alpha_{2}=0$, the regression continues up to the classical problem.

### 4.2. Plane Section Assumption

For the pure bending problem, the rotation of a vertical element of the cross section, β, may be obtained from Equation (61),

(80) $$\beta =\frac{\partial u}{\partial z}=\frac{M}{E_{0}b(A_{2}^{+}+A_{2}^{-})}(x-l).$$

It is obvious that the rotation is not dependent on *z*, which shows that for the pure bending problem, the plane section assumption is surely satisfied. However, for the lateral-force bending problem, the rotation may be obtained from Equation (78), respectively, for the tensile area

(81) $$\beta =\frac{\partial u^{+}}{\partial z}=\frac{P[(1+\mu )h(12h\alpha_{1}-12\alpha_{1}^{2}z)+3\mu \alpha_{1}^{3}z^{2}+3\alpha_{1}^{3}x^{2}-12(1+\mu )\alpha_{1}he^{\alpha_{1}(h_{1}-z)/h}\left(h-h_{1}\alpha_{1}\right)]}{6E_{0}b\alpha_{1}^{3}(A_{2}^{+}+A_{2}^{-})}-a^{+},$$

and for the compressive area

(82) $$\beta =\frac{\partial u^{-}}{\partial z}=\frac{P[(1+\mu )h(12h\alpha_{2}-12\alpha_{2}^{2}z)+3\mu \alpha_{2}^{3}z^{2}+3\alpha_{2}^{3}x^{2}-12(1+\mu )\alpha_{2}he^{-\alpha_{2}(h_{2}+z)/h}(h+h_{2}\alpha_{2})]}{6E_{0}b\alpha_{2}^{3}(A_{2}^{+}+A_{2}^{-})}-a^{-}.$$

It is readily seen that the rotation is now the function of *z*. This means that on any cross section, a vertical element under bending will deviate from the original vertical direction and the deviated value varies with the distance from the neutral layer, i.e., *z*. Consequently, for the lateral-force bending problem the plane section assumption no longer holds. Moreover, unlike the pure bending problem, the rotation will not continuously develop at the neutral layer due to the difference in tension and compression.

### 4.3. Bimodular Effect on Stress and Displacement

The bimodular effect on stress and displacement may be analyzed by the use of the analytical results obtained. To avoid the inconvenience introduced by the dimension of physical quantities, besides Equation (23), we adopt the following dimensionless manner:

(83) $$\begin{array}{l} m=\frac{M}{E_{0}h^{3}},\;p=\frac{P}{E_{0}h^{2}},\;a=\frac{A_{2}^{+}+A_{2}^{-}}{h^{3}},\;\zeta =\frac{z}{h},\;\eta =\frac{x}{l},\;\\ s^{+/-}=\frac{\sigma_{x}^{+/-}b}{E_{0}h},\;t^{+/-}=\frac{\tau_{zx}^{+/-}b}{E_{0}h},\;u^{*}=\frac{ub}{l^{2}},\;w^{*}=\frac{wb}{l^{2}},\; \end{array}.$$

The two-dimensional solution for stress and displacement under pure bending, i.e., Equations (49) and (61), may be changed as, respectively,

(84) $$s^{+}=\frac{m}{a}\zeta e^{\alpha_{1}\zeta },\;\mathrm{for}\;0\leq \zeta \leq H_{1};\;s^{-}=\frac{m}{a}\zeta e^{\alpha_{2}\zeta },\;\mathrm{for}\;-H_{2}\leq \zeta \leq 0,$$

and

(85) $$u^{*}=\frac{m}{a}\frac{h}{l}(\eta -1)\zeta ,w^{*}=-\frac{m}{2a}[\eta^{2}+\mu {\left(\frac{h}{l}\right)}^{2}\zeta^{2}-2\eta +1].$$

The above dimensionless displacement is helpful for analyzing the approximation degree from a two-dimensional solution to a one-dimensional one. We note that there exists a common factor h/l in the expressions of $u^{*}$ and $w^{*}$. If a typical shallow beam is considered here, the ratio of the beam height to span length will be much less than 1, i.e., h/l≪1; this makes the magnitude of the $u^{*}$ value much less than the value of $w^{*}$. Thus, in one-dimensional beam theory the horizontal displacement $u^{*}$ is generally neglected without much error. On the other hand, if h/l≪1, also the term ${\left(h/l\right)}^{2}\ll 1$ and 0<μ<0.5 for common materials; thus, the second term $\mu {\left(h/l\right)}^{2}\zeta^{2}$ in $w^{*}$ may be neglected comparing to other items. This yields

(86) $$u^{*}=0,\;w^{*}=-\frac{m}{2a}{\left(\eta -1\right)}^{2},$$

which is exactly the dimensionless one-dimensional solution for displacement.

Similarly, the two-dimensional solution for stress under lateral-force bending, i.e., Equation (77), may be changed as

(87) $$\begin{array}{l} s^{+}=\frac{p}{a}\frac{l}{h}\eta \zeta e^{\alpha_{1}\zeta },\;\mathrm{for}\;0\leq \zeta \leq H_{1};\;s^{-}=\frac{p}{a}\frac{l}{h}\eta \zeta e^{\alpha_{2}\zeta },\;\mathrm{for}\;-H_{2}\leq \zeta \leq 0 \end{array}$$

(88) $$\begin{array}{l} t^{+}=\frac{p}{a}\left[\left(\frac{1}{\alpha_{1}^{2}}-\frac{\zeta }{\alpha_{1}}\right)e^{\alpha_{1}\zeta }-\left(\frac{1}{\alpha_{1}^{2}}-\frac{H_{1}}{\alpha_{1}}\right)e^{\alpha_{1}H_{1}}\right],\;\mathrm{for}\;0\leq \zeta \leq H_{1}\\ t^{-}=\frac{p}{a}\left[\left(\frac{1}{\alpha_{2}^{2}}-\frac{\zeta }{\alpha_{2}}\right)e^{\alpha_{2}\zeta }-\left(\frac{1}{\alpha_{2}^{2}}+\frac{H_{2}}{\alpha_{2}}\right)e^{-\alpha_{2}H_{2}}\right],\;\mathrm{for}\;-H_{2}\leq \zeta \leq 0 \end{array}.$$

**Figure 3:**
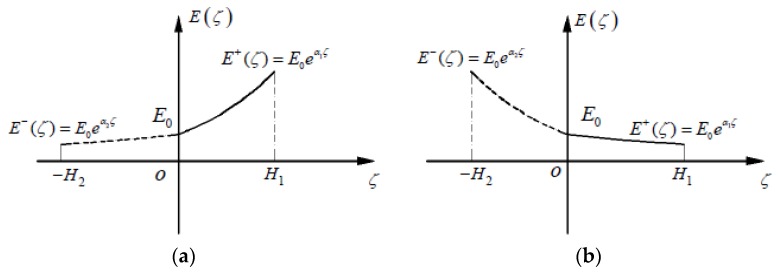
Variation of E(ζ) with the thickness direction. (**a**) $\alpha_{1}>0$ and $\alpha_{2}>0$; (**b**) $\alpha_{1}<0$ and $\alpha_{2}<0$.

Considering the characteristics of the grade function $E^{+}(\zeta )=E_{0}e^{\alpha_{1}\zeta }$ where $0\leq \zeta \leq H_{1}$ and $E^{-}(\zeta )=E_{0}e^{\alpha_{2}\zeta }$ where $-H_{2}\leq \zeta \leq 0$, it is easily found from Figure 3 that if the grade indexes $\alpha_{1}>0$ and $\alpha_{2}>0$, $E^{+}(\zeta )>E^{-}(\zeta )$ holds; if $\alpha_{1}<0$ and $\alpha_{2}<0$, $E^{+}(\zeta )<E^{-}(\zeta )$ holds; obviously, $\alpha_{1}=\alpha_{2}=0$ corresponds to the classical problem. Therefore, 13 representative examples concerning the taken values of $\alpha_{1}$ and $\alpha_{2}$ are selected, including ±0.5, ±1.0, and ±2.0. Some relative parameters, including $H_{1}$ and $H_{2}$ (from Equation (24)) and *a* (from Equation (83)), are computed and listed in Table 2.

From Table 2, it is easily found that for $E^{+}(\zeta )>E^{-}(\zeta )$, as the values of $\alpha_{1}$ and $\alpha_{2}$ increase, the tensile height decreases while the compressive height increases, which means that the neutral axis is tending downward (see Figure 1 and Figure 2, *z* axis is down); for $E^{+}(\zeta )<E^{-}(\zeta )$, as the absolute values of $\alpha_{1}$ and $\alpha_{2}$ increase, the tensile height increases while the compressive height decreases, which means the neutral axis is tending upward. Besides this, we also note another interesting phenomenon, which is that due to the characteristic of an exponential function, the heights in tension and compression $H_{1}$ and $H_{2}$ are exactly reversed in some cases, including groups (a) and (g), (b) and (h), (c) and (i), and (e) and (k). For the values of *a*, they are the same as in the combinations above.

**Figure 4:**
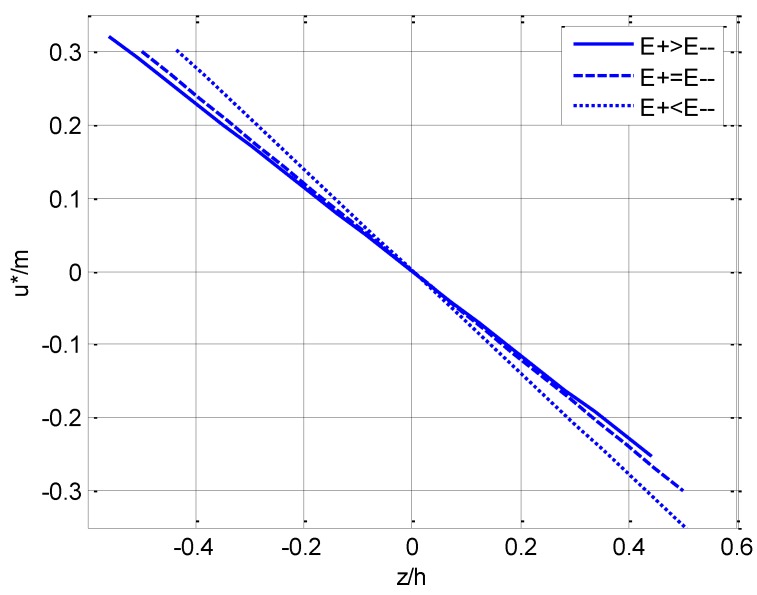
Variation of displacement $u^{*}$ at midspan (η=0.5) with ζ(=z/h) in three cases.

**Figure 5:**
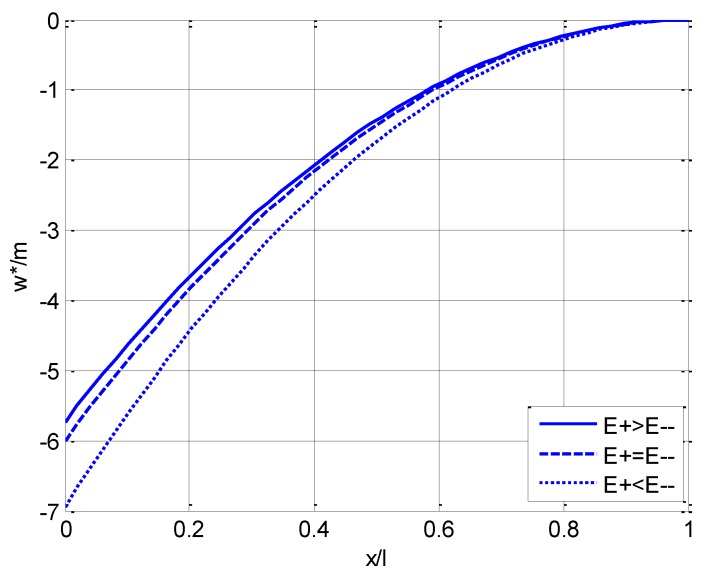
Variation of deflection $w^{*}$ of the neutral layer (ζ=0) with η(=x/l) in three cases.

If the midspan displacement (i.e., x=l/2 or η=0.5) of a beam under pure bending is considered, $u^{*}$ in Equation (85) may be changed as

(89) $$\frac{u^{*}}{m}=-\frac{\zeta }{20a},$$

where *h/l* is taken as 1/10. For the main three types of cases listed in Table 2, i.e., the representative groups (d), (f), and (j), the varying curves of $u^{*}/m$ with ζ(=z/h) as well as the deflection curve of the neutral layer (ζ=0, see $w^{*}/m$ in Equation (86)) with η(=x/l) are plotted in Figure 4 and Figure 5, respectively, in which the solid lines correspond to the case of $E^{+}(\zeta )>E^{-}(\zeta )$, the dashed lines correspond to the case of $E^{+}(\zeta )=E^{-}(\zeta )$, and the dotted lines correspond to the case of $E^{+}(\zeta )<E^{-}(\zeta )$.

**Figure 6:**
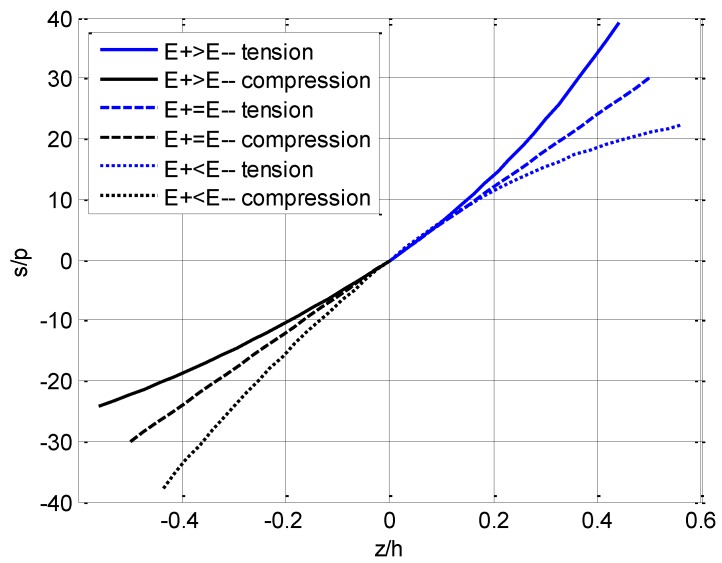
Variation of bending stress s at midspan (η=0.5) with ζ(=z/h) in three cases.

**Figure 7:**
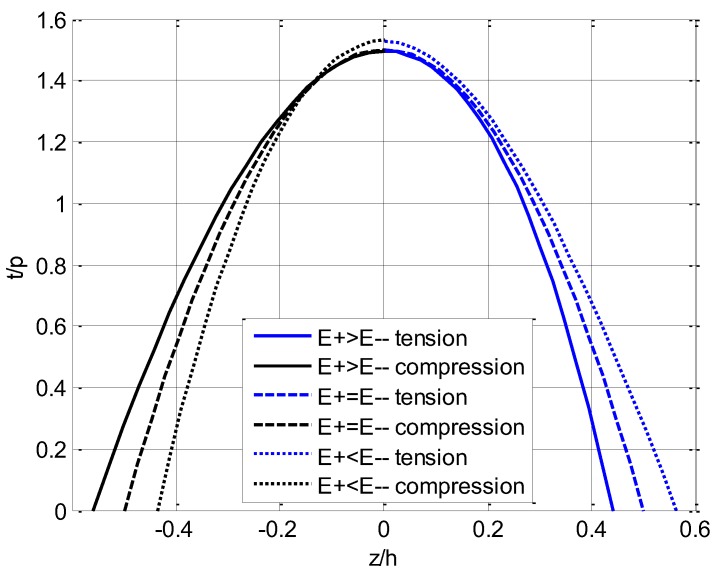
Variation of shearing stress t with ζ(=z/h) in three cases.

Similarly, we may use the midspan stress formulas (η=0.5) of a beam under lateral-force bending to analyze the bimodular effect on the bending stress and shearing stress. Thus, Equation (87) is changed as

(90) $$\frac{s^{+}}{p}=\frac{5}{a}\zeta e^{\alpha_{1}\zeta },\;\mathrm{for}\;0\leq \zeta \leq H_{1};\;\frac{s^{-}}{p}=\frac{5}{a}\zeta e^{\alpha_{2}\zeta },\;\mathrm{for}\;-H_{2}\leq \zeta \leq 0$$

where l/h=10. For the main three cases listed in Table 2, the variation of stresses with ζ(=z/h) are plotted in Figure 6 and Figure 7, in which the shearing stress curve t/p is directly from Equation (88).

We should note such a fact that since the neutral layer is established on the *x* axis beforehand, the dividing line between tension and compression is always on ζ=0, which may be easily seen from Figure 4, Figure 6 and Figure 7. Figure 4 shows that the horizontal displacement varies in a linear relation along the direction of the beam thickness as indicated in Equation (89). The maximum horizontal displacement takes place at the edge of the compressive area for $E^{+}(\zeta )>E^{-}(\zeta )$ and at the edge of the tensile area for $E^{+}(\zeta )<E^{-}(\zeta )$, while the maximum displacement is equal for $E^{+}(\zeta )=E^{-}(\zeta )$. Figure 5 shows that, for any point on the neutral layer, the deflection value when $E^{+}(\zeta )>E^{-}(\zeta )$ is always less than the corresponding value when $E^{+}(\zeta )<E^{-}(\zeta )$.

Figure 6 presents a typical exponent relation of bending stress varying along the direction of the beam thickness. Due to the variation of elastic modulus with the thickness direction, the location at which the maximum stress takes place may be changed. For $E^{+}(\zeta )>E^{-}(\zeta )$, the maximum tensile stress still takes place at the tensile edge of the beam while the maximum compressive stress will take place on a certain level between the compressive edge and the neutral layer; for $E^{+}(\zeta )<E^{-}(\zeta )$, the maximum compressive stress still takes place at the compressive edge of the beam while the maximum tensile stress will take place on a certain level between the tensile edge and the neutral layer; for $E^{+}(\zeta )=E^{-}(\zeta )$, the maximum tensile and compressive stress are equal and take place at the tensile and compressive edges of the beam, respectively, as we expected. This conclusion may be proved by the use of the extreme condition for an analytical solution of bending stress. We take the first-order derivative of bending stress with respect to the thickness direction, *z*, such that,

(91) $$\frac{\partial \sigma_{x}^{+/-}}{\partial z}=\frac{\partial }{\partial z}\frac{M(x)}{b(A_{2}^{+}+A_{2}^{-})}ze^{\alpha_{i}z/h}=\frac{M(x)}{b(A_{2}^{+}+A_{2}^{-})}(e^{\alpha_{i}z/h}+z\frac{\alpha_{i}}{h}e^{\alpha_{i}z/h}),$$

where M(x)=M for pure bending and M(x)=Px for lateral bending. Via extreme conditions $\partial \sigma_{x}^{+/-}/\partial z=0$, we have

(92) $$e^{\alpha_{i}z/h}(1+z\frac{\alpha_{i}}{h})=0,$$

$e^{\alpha_{i}z/h}>0$ permanently holds true, we have

(93) $$z=-\frac{h}{\alpha_{i}},$$

which determines the location at which the maximum tensile or compression stress takes place. By referring to Figure 3, it is obvious that for $E^{+}(\zeta )>E^{-}(\zeta )$, the maximum compressive stress takes place at $z=-h/\alpha_{2}$; for $E^{+}(\zeta )<E^{-}(\zeta )$, the maximum tensile stress takes place at $z=-h/\alpha_{1}$. This phenomenon is quite different from the classical problem.

For the three cases of different moduli in tension and compression, Figure 7 uniformly indicates that the maximum shearing stress takes place at the neutral layer (ζ=0) and takes zero at the top and bottom of the beam. For $E^{+}(\zeta )=E^{-}(\zeta )$, the shearing stress in tension and compression is symmetrical with respect to ζ=0, while for the other two cases the rule does not hold. Moreover, the maximum shearing stress in the case of $E^{+}(\zeta )>E^{-}(\zeta )$ is less than the maximum stress in the case of $E^{+}(\zeta )<E^{-}(\zeta )$.

## 5. Concluding Remarks

In this study, one-dimensional and two-dimensional mechanical models for a functionally graded beam with different moduli in tension and compression were established. The corresponding analytical solutions under pure bending and lateral-force bending were obtained. The following three conclusions can be drawn.

(1) The mechanical models established on the one-dimensional and two-dimensional theory are consistent; the two-dimensional solution may regress to the corresponding one-dimensional solution.

(2) For pure bending problems, the plane section assumption still holds for a bimodular functionally graded beam; for lateral-force bending problems, the plane section assumption holds only in the case of a shallow beam.

(3) The introduction of the bimodular effect and functionally graded characteristic of materials will change the stress and deformation of the structure to some extent. Specifically, the maximum bending stress may take place at a certain level between the neutral layer and edge fibers of the beam, which should be given more attention in the analysis and design of similar structures.

The material considered in this study not only has a functionally graded characteristic but also exhibits different tensile and compressive moduli of elasticity, which further complicates the analysis of similar structures made from these materials. It will be worthwhile considering the plate model adopting classical plate theory for laminate (or higher order theory) to discretize the material properties along the direction of the plate thickness (or here along the beam height).

Moreover, since beams, plates, and shells can all be attributed to, from the point of view of loading and deformation, bending elements under external loads, this work may be extended to the static and dynamic responses of functionally graded beams [27], of functionally graded plates [28], as well as of functionally graded shells [29], in which the bimodular effect of the materials will be incorporated. At the same time, this work may also be extended to an investigation on the existing capabilities and limitations in numerical modeling of fracture problems in functionally graded materials by means of the well-known finite element code ABAQUS [30]. We will study these interesting issues in the future.

## Figures and Tables

**Table 1:** Comparisons among a classical beam, an FGM beam, and a bimodular FGM beam.

Quantities	A Classical Beam	A FGM Beam	A Bimodular FGM Beam
Modulus of elasticity			
E	E=Const.	$E(z)=E_{0}e^{\alpha z/h}$	$E^{+}(z)=E_{0}e^{\alpha_{1}z/h},E^{-}(z)=E_{0}e^{\alpha_{2}z/h}$
Moment of inertia			
$I_{y}$	$\frac{bh^{3}}{12}$	$\int_{A}e^{\alpha z/h}z^{2}dA$	$\begin{array}{l} \int_{0}^{h_{1}}e^{\alpha_{1}z/h}z^{2}bdz+\int_{-h_{2}}^{0}e^{\alpha_{2}z/h}z^{2}bdz\\ =b(A_{2}^{+}+A_{2}^{-}) \end{array}$
Bending stiffness			
D	$EI_{y}$	$E_{0}\int_{A}e^{\alpha z/h}z^{2}dA$	$E_{0}b(A_{2}^{+}+A_{2}^{-})$
Curvature			
$\frac{1}{\rho }$	$\frac{M}{EI_{y}}$	$\frac{M}{E_{0}\int_{A}e^{\alpha z/h}z^{2}dA}$	$\frac{M}{E_{0}b(A_{2}^{+}+A_{2}^{-})}$
Bending stress			
$\sigma_{x}$	$\frac{M}{I_{y}}z$	$\frac{Mz}{\int_{A}e^{\alpha z/h}z^{2}dA}$	$\begin{array}{l} \sigma_{x}^{+}=\frac{M}{b(A_{2}^{+}+A_{2}^{-})}ze^{\alpha_{1}z/h}\\ \sigma_{x}^{-}=\frac{M}{b(A_{2}^{+}+A_{2}^{-})}ze^{\alpha_{2}z/h} \end{array}$
Static moment when computing shearing stress			
$S_{y}$	$\frac{b}{2}\left(\frac{h^{2}}{4}-z^{2}\right)$	$\int_{A}e^{\alpha z/h}zdA$	$\left\{ \begin{array}{ll} S^{+} & =\int_{z}^{h_{1}}e^{a_{1}z/h}zbdz\;\mathrm{for}\;0\leq z\leq h_{1}\\ & =b[(\frac{h^{2}}{a_{1}^{2}}-\frac{hz}{a_{1}})e^{a_{1}z/h}-(\frac{h^{2}}{a_{1}^{2}}-\frac{hh_{1}}{a_{1}})e^{a_{1}h_{1}/h}]\\ S^{-} & =\int_{z}^{-h_{1}}e^{a_{2}z/h}zbdz\;\mathrm{for}\;-h_{2}\leq z\leq 0\\ & =b[(\frac{h^{2}}{a_{2}^{2}}-\frac{hz}{a_{2}})e^{a_{2}z/h}-(\frac{h^{2}}{a_{2}^{2}}+\frac{hh_{2}}{a_{2}})e^{a_{2}h_{2}/h}] \end{array}\right.$
Shearing stress			
$\tau_{xz}$	$\frac{PS_{y}}{I_{y}b}$	$\frac{P\int_{A}e^{\alpha z/h}zdA}{b\int_{A}e^{\alpha z/h}z^{2}dA}$	$\left\{ \begin{array}{l} \tau_{zx}^{+}=\;\mathrm{for}\;0\leq z\leq h_{1}\\ \frac{P}{b(A_{2}^{+}+A_{2}^{-})}[(\frac{h^{2}}{a_{1}^{2}}-\frac{hz}{a_{1}})e^{a_{1}z/h}-(\frac{h^{2}}{a_{1}^{2}}-\frac{hh_{1}}{a_{1}})e^{a_{1}h_{1}/h}],\\ \tau_{zx}^{+}=\;\mathrm{for}\;-h_{2}\leq z\leq 0\\ \frac{P}{b(A_{2}^{+}+A_{2}^{-})}[(\frac{h^{2}}{a_{2}^{2}}-\frac{hz}{a_{2}})e^{a_{2}z/h}-(\frac{h^{2}}{a_{2}^{2}}-\frac{hh_{2}}{a_{2}})e^{a_{2}h_{2}/h}], \end{array}\right.$

**Table 2:** Numerical values of $H_{1}$, $H_{2}$, and *a* in different cases.

Cases	Groups	$\alpha_{1}$	$\alpha_{2}$	$H_{1}$	$H_{2}$	a
$E^{+}(\zeta )>E^{-}(\zeta )$	(a)	1.0	2.0	0.3725	0.6275	0.0560
	(b)	2.0	1.0	0.3859	0.6141	0.0836
	(c)	1.0	1.0	0.4180	0.5820	0.0762
	(d)	1.0	0.5	0.4399	0.5601	0.0872
	(e)	0.5	0.5	0.4585	0.5415	0.0815
$E^{+}(\zeta )=E^{-}(\zeta )$	(f)	0	0	1/2	1/2	1/12
$E^{+}(\zeta )<E^{-}(\zeta )$	(g)	−2.0	−1.0	0.6275	0.3725	0.0560
	(h)	−1.0	−2.0	0.6141	0.3859	0.0836
	(i)	−1.0	−1.0	0.5820	0.4180	0.0762
	(j)	−1.0	−0.5	0.5638	0.4362	0.0720
	(k)	−0.5	−0.5	0.5415	0.4585	0.0815
